# The Thermal Death Point of Septic Organisms

**Published:** 1878-11

**Authors:** 


THE THERMAL DEATH POINT OF SEPTIC ORGANISMS.—Some very careful experiments on this subject have lately been rehearsed to the Royal Society by the Rev. W. N. Dallinger. He founc septic organisms living after exposure to a heat of 250° Fahrenheit. He proceeds in his narrative:

I followed this with four more experiments, separately and successively made. Two of them were at a temperature of 248°, and two at 250° F. In both of the former, at the end of nine or ten hours, the complete organism in full vigor could be seen; and in one of the cases it was discovered still in the condition shown at Fig. 20, Plate 2, and watched until the organisms had attained the condition indicated in Fig. 24, Plate 2.

But in the two latter instances (heated up to 250°) the living form did not appear during the six days following, although repeatedly looked for.

I concluded, therefore, that the temperature of 250° F. was the limit of endurance which the spore of this form could bear by this method of heating.

Boiling water, therefore, would not destroy these germs. *Cin. Lancet and Clinic.*

